# Image-Based Automated Recognition of 31 Poaceae Species: The Most Relevant Perspectives

**DOI:** 10.3389/fpls.2021.804140

**Published:** 2022-01-26

**Authors:** Michael Rzanny, Hans Christian Wittich, Patrick Mäder, Alice Deggelmann, David Boho, Jana Wäldchen

**Affiliations:** ^1^Department of Biogeochemical Integration, Max Planck Institute for Biogeochemistry, Jena, Germany; ^2^Data-intensive Systems and Visualisation, Technische Universität Ilmenau, Ilmenau, Germany; ^3^Faculty of Biological Sciences, Friedrich Schiller University, Jena, Germany

**Keywords:** deep learning, machine learning, accuracy, Poaceae, plant perspective, image recognition, fine-grained image classification, automated plant identification

## Abstract

Poaceae represent one of the largest plant families in the world. Many species are of great economic importance as food and forage plants while others represent important weeds in agriculture. Although a large number of studies currently address the question of how plants can be best recognized on images, there is a lack of studies evaluating specific approaches for uniform species groups considered difficult to identify because they lack obvious visual characteristics. Poaceae represent an example of such a species group, especially when they are non-flowering. Here we present the results from an experiment to automatically identify Poaceae species based on images depicting six well-defined perspectives. One perspective shows the inflorescence while the others show vegetative parts of the plant such as the collar region with the ligule, adaxial and abaxial side of the leaf and culm nodes. For each species we collected 80 observations, each representing a series of six images taken with a smartphone camera. We extract feature representations from the images using five different convolutional neural networks (CNN) trained on objects from different domains and classify them using four state-of-the art classification algorithms. We combine these perspectives *via* score level fusion. In order to evaluate the potential of identifying non-flowering Poaceae we separately compared perspective combinations either comprising inflorescences or not. We find that for a fusion of all six perspectives, using the best combination of feature extraction CNN and classifier, an accuracy of 96.1% can be achieved. Without the inflorescence, the overall accuracy is still as high as 90.3%. In all but one case the perspective conveying the most information about the species (excluding inflorescence) is the ligule in frontal view. Our results show that even species considered very difficult to identify can achieve high accuracies in automatic identification as long as images depicting suitable perspectives are available. We suggest that our approach could be transferred to other difficult-to-distinguish species groups in order to identify the most relevant perspectives.

## 1. Introduction

Automated species identification is becoming an important and widely used tool to monitor the occurrence of species across a wide taxonomic range (Durso et al., 2021; Høye et al., 2021; Joly et al., 2021; Mahecha et al., 2021). While a lot of literature on automated identification of plants in general is published, little is known about how well certain difficult taxonomic groups are recognized by automated identification algorithms and how this might be improved. Most notably, species belonging to the plant family of Poaceae are all characterized by a uniform visual appearance, making it a major challenge in image based plant identification. About 12,000 species and 780 genera of Poaceae are described (Christenhusz and Byng, 2016; Soreng et al., 2017) which ranks them among the most diverse plant families worldwide. Species of this family are circumpolar distributed and are of great ecologic and economic value. Many species are cultivated as important food and forage plants while others are frequent and abundant weeds in various crops (Schroeder et al., 1993). With only a few exceptions all Poaceae species are characterized by a unique set of characters that allows an easy attribution of individuals as members of the this family (). This more or less uniform morphology leads to the common perception of “grass” as a single species in the public (Jäkel and Schaer, 2004; Thomas, 2019). The sometimes very subtle differences between species or even genera can only be recognized by careful examination, especially if no flowers are present.

Automated identification applications achieve moderate to high accuracies in both, plant recognition from images (Wäldchen et al., 2018; Joly et al., 2021) and *in vivo* in the field (Bonnet et al., 2018; Jones, 2020; Pärtel et al., 2021). Reliable identifications are crucial for the credibility of the collected data and also for professional users such as farmers, foresters or teachers. However, detailed evaluations of identification accuracy across broader taxonomic groups have identified Poaceae to be among the families achieving lowest accuracies (Rzanny et al., 2019; Pärtel et al., 2021). In order to generate valid plant species distribution data *via* automated plant identification apps (e.g., Bonnet et al., 2020; Mahecha et al., 2021) it is of vital importance to enable users to reliably differentiate Poaceae species which are often not recognized on species level. Poaceae species are ubiquitous, often dominate entire landscapes (Veen et al., 2009) and their occurrence and distribution provide invaluable information on the condition and development of the habitat (e.g., Diekmann et al., 2019). Experiments to evaluate fine-grained classification within a group of visually very similar plant species have been performed e.g., for Chenopodiaceae, which represent another plant family with mainly wind- or self pollinated and inconspicuous flowers (Heidary-Sharifabad et al., 2021). The developed classifier is able to differentiate between 30 species of Chenopodiaceae with an accuracy of about 90%. The study by Golzarian and Frick (2011) was an earlier attempt to distinguish seedlings of ryegrass and bromegrass from wheat based on a combination of color, texture and shape feature vectors which were represented as three descriptors derived from principal component analysis. The authors were able to distinguish ryegrass from wheat with an accuracy of 88% and bromegrass from wheat with an accuracy of 83%. Another recent study (Rzanny et al., 2019) distinguished 12 Poaceae species as part of a larger species subset with an accuracy of 90% when all considered perspectives were fused. Combinations of only some of these perspectives yielded slightly better results (up to 92.5%) and the authors noted that the utilized perspectives were not sufficient to reliably identify the species under consideration. Images of reproductive plant parts are generally more often identified correctly than non-reproductive parts such as leaves or stems (Rzanny et al., 2019; Pärtel et al., 2021). We expect the classification of images depicting inflorescences to achieve better results than images from vegetative parts. However, it is often not sufficient for a valid identification to solely rely on the images of flowers or inflorescences. Especially for a number of congeneric species, images of more specific characters might be required to allow a reliable identification.

An important aspect of this study is to assess the predictive value of vegetative parts of Poaceae species which are present for longer time periods throughout the year. Here, we consider images depicting the collar region, the adaxial and abaxial parts of the lamina and the nodes, which all might display species-specific characters. However, it is unknown what kind of perspective and which region of a Poaceae species provides the most relevant information in a single image or which combination of multiple perspectives allows a reliable identification of the species even in the absence of flowers. In order to draw general conclusions from our experiment we decided to distribute the image analysis over a range of deep neuronal networks for feature extraction and the classification of these feature vectors over multiple methods as well. If certain perspectives provide important information for the determination of a species, this perspective should also achieve a high relative accuracy across different feature extraction and classification algorithms. Although we expect a CNN trained on plant images to achieve higher absolute values of accuracy, we expect the relative rank of the different perspectives to remain comparable across the array of methods if the results are not influenced by overfitting of certain highly specialized CNNs.

Consequently, the aims of this study are fourfold: (1) We evaluate six image perspectives regarding the information they convey for Poaceae species identification. (2) We seek to find the most accurate combination of image perspectives for the identification of Poaceae species. (3) We assess how the accuracy of each perspective differs across the range of used feature extraction algorithms and classifiers. (4) We explore the potential of identifying Poaceae species without the presence of flowers.

## 2. Materials and Methods

### 2.1. Poaceae Morphology

**Figure 1:**
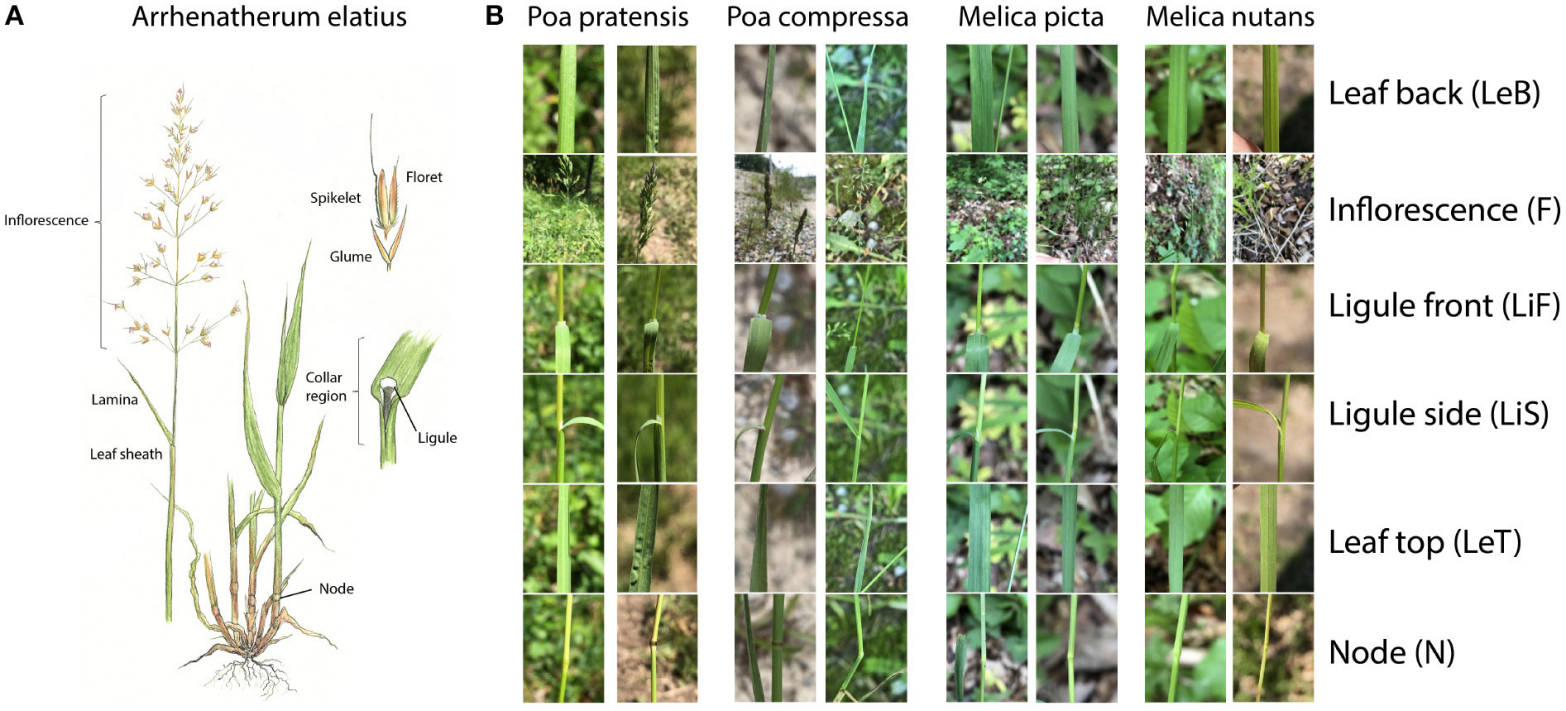
**(A)** Illustration of the structure of a typical Poaceae species: *Arrhenatherum elatius*; drawing by Rita Lüder (Lüder and Lüder, 2011). **(B)** Two full observations per species depicted for four different species. The perspective names and their abbreviations are denoted on the right.

All Poacaeae, and therefore also the largest subfamily Pooideae, which all considered species belong to, are characterized by common morphological characters which are responsible for the uniform appearance of different species (Figure 1). The stems (culms) are round with solid nodes and hollow internodes. Leaf position is distichous and alternate and they are attached to the nodes of the culm. The leaves are lineal with parallel veins and consist of a culm-enclosing sheath at the lower part and a free lamina at the upper part. At the junction of sheath and lamina a translucent, membranous outgrowth is located. This structure is referred to as ligule. It can also be developed as a fringe of hairs in some genera and in rare cases it is missing. Some species additionally exhibit a pair of claw-like or ear-like appendages at the base of the lamina (auricles). The transition of sheath and lamina is called collar region and is highly indicative for species identification through often unique combinations of characters. Poaceae have reduced flowers. Their inflorescences can be grouped as panicles, spikes or racemes, depending on the presence and branching of the pedicels. The inflorescence is composed of spikelets. They represent the flowering unit and are covered by two glumes. Each spikelet in turn is composed of one or more florets which form the reproductive unit. The flower itself is covered by two bracts (palea + lemma) where the midrib of the latter may or may not be prolonged into a fibrous bristle termed awn.

### 2.2. Image Acquisition

We collected 80 observations for each of the studied 31 Poaceae species (Table 1) in two consecutive years (2019 and 2020). All images analyzed in this study were collected in different regions of Germany using the Flora Capture app (Boho et al., 2020). This smartphone app is designed to collect plant images from several perspectives. Species taxonomy in this app basically follows (Roskov et al., 2019) but in some cases very similar species are summarized to aggregates (Table 1). The following six perspectives were photographed at minimal focusing distance: (1) Node (N) - depicts the culm node from lateral position. (2) - Inflorescence (F) - a lateral image of the entire inflorescence. If the entire inflorescence exceeded the image at minimal focusing distance, the distance was increased until the entire inflorescence could be photographed. (3) Leaf back (LeB)-abaxial side of a leaf at medium length and in vertical direction. (4) Leaf top (LeT)-adaxial side of a leaf at medium length and in vertical direction. (5) Ligule side (LiS)-an image of the ligule in lateral perspective. If the leaf was also in vertical position and would conceal the ligule, the leaf was slightly pulled down to ensure visibility of the ligule. (6) Ligule front (LiF) - image of the ligule in frontal position (i.e from a position of the lamina). Again, the leaf was slightly pulled down to ensure visibility of the ligule if necessary. Whenever possible, more than one species was sampled at a each location. All images were obtained from flowering individuals by five different persons using a range of smartphone models (iOS+Android). Exemplary observations of four different species are shown in Figure 1.

**Table 1:** List of all species that were used in the identification experiment.

**Full species name**	**Abbreviation**	**Code**
*Agrostis capillaris* L.	Agr_cap	1
*Agrostis stolonifera* L.	Agr_sto	2
*Alopecurus pratensis* L.	Alo_pra	3
*Anthoxanthum odoratum* L.	Ant_odo	4
*Avenula pubescens* (Huds.) Dumort.	Ave_pup	5
*Arrhenatherum elatius* (L.) P.Beauv. ex J.Presl & C.Presl.	Arr_ela	6
*Brachypodium pinnatum* (L.) P.Beauv.	Bra_pin	7
*Brachypodium sylvaticum* (Huds.) P.Beauv.	Bra_syl	8
*Bromus inermis* Leyss.	Bro_ine	9
*Bromus erectus* Huds.	Bro_ere	10
*Bromus hordeaceus* agg.	Bro_hor	11
includes *Bromus hordeaceus* L.		
includes *Bromus lepidus* Holmb.		
*Bromus ramosus* agg.	Bro_ram	12
includes *Bromus ramosus* Huds.		
includes *Bromus benekenii* (Lange) Trimen)		
*Bromus sterilis* L., nom. cons.	Bro_ste	13
*Dactylis glomerata* L.	Dac_glo	14
*Elymus caninus* L.	Ely_can	15
*Elymus repens* L. Gould	Ely_rep	16
*Festuca altissima* All.	Fes_alt	17
*Holcus lanatus* L.	Hol_lan	18
*Hordelymus europaeus* (L.) Jess. ex Harz	Hor_eur	19
*Lolium perenne* L.	Lol_per	20
*Lolium giganteum* (L.) Darbysh.	Lol_gig	21
*Melica nutans* L.	Mel_nut	22
*Melica picta* K.Koch	Mel_pic	23
*Milium effusum* L.	Mil_eff	24
*Phleum pratense* L.	Phl_pra	25
*Poa compressa* L.	Poa_com	26
*Poa nemoralis* L.	Poa_nem	27
*Poa pratensis* L.	Poa_pra	28
*Poa trivialis* L.	Poa_triv	29
*Sesleria caerulea* (L.) Ard.	Ses_cae	30
*Trisetum flavescens* (L.) P.Beauv.	Tri_fla	31

*Species names are used according to Catalog of Life (Roskov et al., 2019)*.

### 2.3. Feature Extraction

Since our dataset consists of a comparatively small number of samples, we approach the expected difficulties of training a classification model with a high number of parameters end-to-end, i.e., overfitting, by separating feature learning and classification tasks. Our pipeline therefore includes two stages: feature extraction, for which we compare the use of different neural networks, trained on data from different problem domains, and supervised classification using a number of well-established algorithms. In the feature extraction stage we project the high-dimensional data of our Poaceae observation images into a lower-dimensional feature space more convenient for classification.

We compare different state-of-the art architectures of deep convolutional neural networks, pre-trained on datasets from various domains, using the feature maps from their final layer as representation to train classifiers on. The goal is to evaluate how well features learned on different problems can be transferred to our independent classification problem. We use the following three architectures of deep convolutional neural networks in our experiments. Inception-v3 (Szegedy et al., 2016) is a 42-layer convolutional neural network that builds on Inception modules, each applying multiple differently-sized convolution filters and pooling operations to the same input in parallel. The network has 23.8M trainable parameters and an input resolution of 299x299 pixels. ResNet (He et al., 2016) uses identity shortcut connections to tackle the problem of vanishing gradients in deep networks. The variant ResNet-101 is 101 layers deep, has 44.5M trainable parameters and operates on images of 224 x 224 pixels. NASNet (Zoph et al., 2018) is a convolutional neural network for which the architecture of the convolutional layers themselves has been optimized in an automated process instead of being designed by experts. The specific version we use has 88.9M trainable parameters and takes input images with a resolution of 331 x 331 pixels. Our observations images are resized to the network's respective input resolution before feature extraction. The neural networks have been trained for supervised classification tasks on the following datasets:

- Open Images (Krasin et al., 2017). The dataset consists of 9.4M labeled training images of a great variety of objects, plants, animals etc. taken in different surroundings without any systematic process that were originally uploaded by users of the image-hosting website Flickr under CC-BY license. They span 5K classes which the authors consider trainable based on the number of human-verified class labels.
- Leafsnap (Kumar et al., 2012). The dataset consists of 25K labeled training images depicting leaves from 184 species of trees from the Northeastern United States. Most images were taken of pressed leaves front- and backlit under controlled lab conditions with uniform background, supplemented by less than 10 percent of field images taken by mobile devices in outdoor environments.
- Birdsnap (Berg et al., 2014). The dataset consists of 50K labeled images of 500 species of birds common in North America. The images show birds in natural surroundings and were taken under various conditions.
- PlantCLEF 2016 (Goëau et al., 2016). The dataset consists of 113K images of 1K species of trees, herbs and ferns distributed in West European regions. Images depict plants under a wide variety of conditions in different surroundings and were taken by different users on their mobile devices.
- Flora Incognita (Mäder et al., 2021). The dataset consists of more than 1M images of 4.8K plant taxa common in Western Europe. It comprises user-contributed images taken from well-defined perspectives *via* the Flora Incognita app (Boho et al., 2020) as well as images taken by experts in the field of botany. Among the taxa included are multiple species of Poaceae.

### 2.4. Image Classification and Evaluation

**Figure 2:**
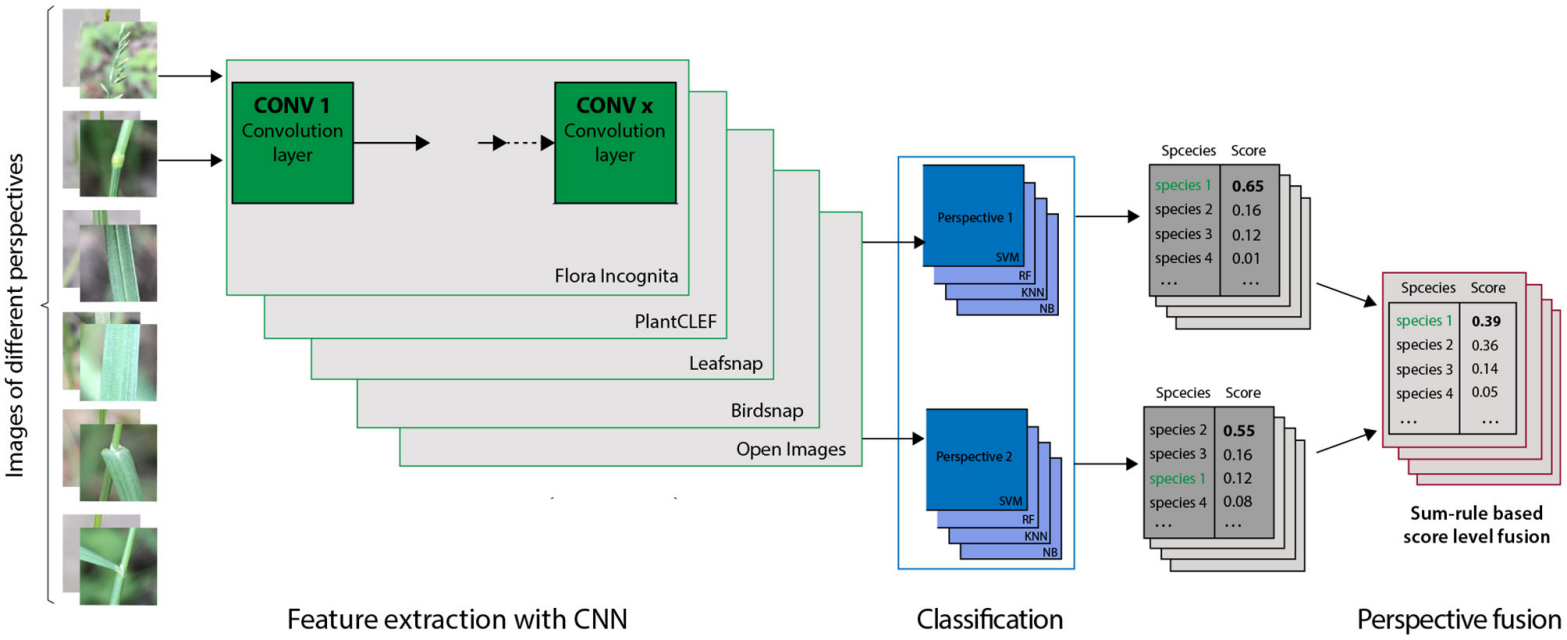
General approach for feature extraction, classification and score fusion used in this study. We used each of the five CNNs (details in the text) to extract features from all images. In the following four different classifiers are trained on the feature vectors of same subset of training images separately for each perspective. The resulting scores for the single perspectives were than fused *via* sum rule, i.e., as the arithmetic mean of the scores for this species for the considered combination of perspectives. The steps were repeated for all CNN - Classifier combinations.

We trained four widely used and established classifiers (Zhang et al., 2017) on the feature vectors extracted by five CNNs from our image data (Figure 2). These five CNNs specifically are Inception-v3 for the Leafsnap, Birdsnap and PlantCLEF datasets, NASNet for Flora Incognita and ResNet-101 for OpenImages. Our dataset was split into a training (75%) and a test (25%) subset with the same split used for all subsequent experiments to calculate classification accuracy. Splitting was stratified by species to ensure that the number of images for training (65) and test (15) were the same for each species. All classifiers were trained and tuned within the caret framework (Kuhn, 2021) in R 4.1.1 (R Core Team, 2020) using the defaults for each classifier but allowing a greater number of parameter combinations (ten instead of three) to be evaluated for model tuning. We used bootstrap resampling (25 iterations) to evaluate the accuracy of the classifier in order to find the best tuning parameters for each classifier. Accuracy was calculated as the percentage of correctly identified images for each species and as the average across all species (recall). We use the following algorithms for classification:

- Support vector machines (SVM) (Cortes and Vapnik, 1995) find an optimal linear hyperplane that separates the classes in the feature space. SVMs are known to be reliable, robust and well-performing learning models (Zhang et al., 2017). We used an SVM classifier with a linear kernel provided by package e1071 (Meyer et al., 2020).
- Random forests (RF) (Breiman, 2001) are ensembles of decision trees, each individually trained on randomly drawn samples of the dataset *via* bootstrap aggregation, thereby generating multiple uncorrelated models whose predictions are combined through voting. We used the implementation provided by package ranger (Wright and Ziegler, 2017)
- The k-Nearest Neighbors (KNN) algorithm (Altman, 1992) assigns a sample's class membership based on the majority class of its *k* nearest neighbors in feature space. We used the implementation provided by package class (Venables and Ripley, 2002)
- Naive Bayes (NB) classifiers use Bayes' rule to estimate the probability of new data belonging to each of the possible classes in a given dataset, thereby assuming independence and gaussian distribution of the descriptors. We used the implementation provided by package naivebayes (Majka, 2019).

To combine the predicted results for multiple perspectives we use score-level fusion based on a simple (normalized) sum rule, an easily comprehensible method that allows for a straightforward interpretation of the results. The fused score *S* over the set *P* of selected perspectives *p*∈*P* is calculated as the sum of the individual scores *s*_*p*_:


$$\begin{array}{l} S=\underset{p\in P}{\sum }\frac{s_{p}}{\left|P\right|} \end{array}$$

## 3. Results

### 3.1. Single Perspectives

The Top-1 accuracies for the individual perspectives averaged across all species range from 87.5 to 26% (inflorescence), 75.3 to 17.2% (ligule front), 70.1 to 18.2% (ligule side), 64.9 to 17.6% (node), 63.7 to 13.1% (leaf back), and 62.3 to 17.6% (leaf top) (**Figure 8**). In general, the features derived from the Flora Incognita CNN combined with an SVM classifier always achieve the highest accuracies, while the Open Images features combined with the Naive Bayes classifier always achieve the lowest accuracies for all single perspectives. This also holds true for the accuracies of almost all different fused combinations. For two combinations (N_F_LiS and N_F_LeB_LiS) the random forest classifier performs slightly better on the Flora Incognita feature vectors than SVM. The inflorescence perspective always achieves the highest accuracy no matter which features are used. The different feature sources maintain a consistent ranking irrespective of the classifier used. Flora Incognita achieves the best accuracies, followed by PlantCLEF, Birdsnap, Leafsnap and Open Images (**Figure 8**). The difference in accuracy between the inflorescence and the remaining perspectives seem to decrease in this order as well.

### 3.2. Perspective Combinations

The accuracy for the inflorescence perspective alone in the best-performing feature extractor-classifier combination (Flora Incognita + SVM) is 87.5% and can be increased by 8.6–96.1% through a combination of all six perspectives (**Figure 5** and Table 2). If considering only those images depicting vegetative plant parts, i.e., excluding inflorescence, the improvement from the best individual perspective (ligule front; 75.3%) to a combination of all five (90.3%) is 15% (**Figure 6** and Table 2). The ranking of the classifiers is largely the same across the entire array of combinations (**Figures 6**–**8**). Also, the differences in usefulness between features from different neural networks are only of quantitative instead of qualitative nature. The same perspective combinations that achieve high accuracies with Flora Incognita features (e.g., N_LiF) also achieve high accuracies with features from the remaining extractors. On the other hand, combinations that perform comparably poorly in Flora Incognita (e.g., LeT_LeB) are also performing poorly with features derived from the other networks (**Figure 6**). In general, the Open Images features not only perform worst in overall accuracies but also show the lowest variation across the perspectives and their combinations (**Figures 6**–**8**).

**Table 2:** Classification results achieved from the best-performing feature extractor (Flora Incognita) and best-performing classifier (SVM).

**Flowers present**		**Vegetative**	
**Perspective name**	**Top-1 Accuracy**	**Perspective name**	**Top-1 Accuracy**
		Node (N)	64.9
**Inflorescence (F)**	87.3		
		Leaf back (LeB)	63.9
		Leaf top (LeT)	62.6
		Ligule side (LiS)	70.3
		**Ligule front (LiF)**	75.1
**Best Combination**	**Top-1 Accuracy**	**Best Combination**	**Top-1 Accuracy**
F_LiS	92.0	LeB_LiF	82.4
N_F_LiF	94.4	N_LeT_LiF	87.1
N_F_LeB_LiF	95.5	N_LeB_LeT_LiF	89.7
N_F_LeT_LeB_LiF	95.3	N_LeB_LeT_LiS_LiF	90.3
N_F_LeT_LeB_LiF_LiS (All)	96.1		

*Top-1 accuracies for individual perspectives and for the best combinations of n perspectives out of all combinations for n=1,…,6. Accuracies are calculated separately depending on the availability of images depicting the inflorescence*.

### 3.3. Species Accurracies

**Figure 3:**
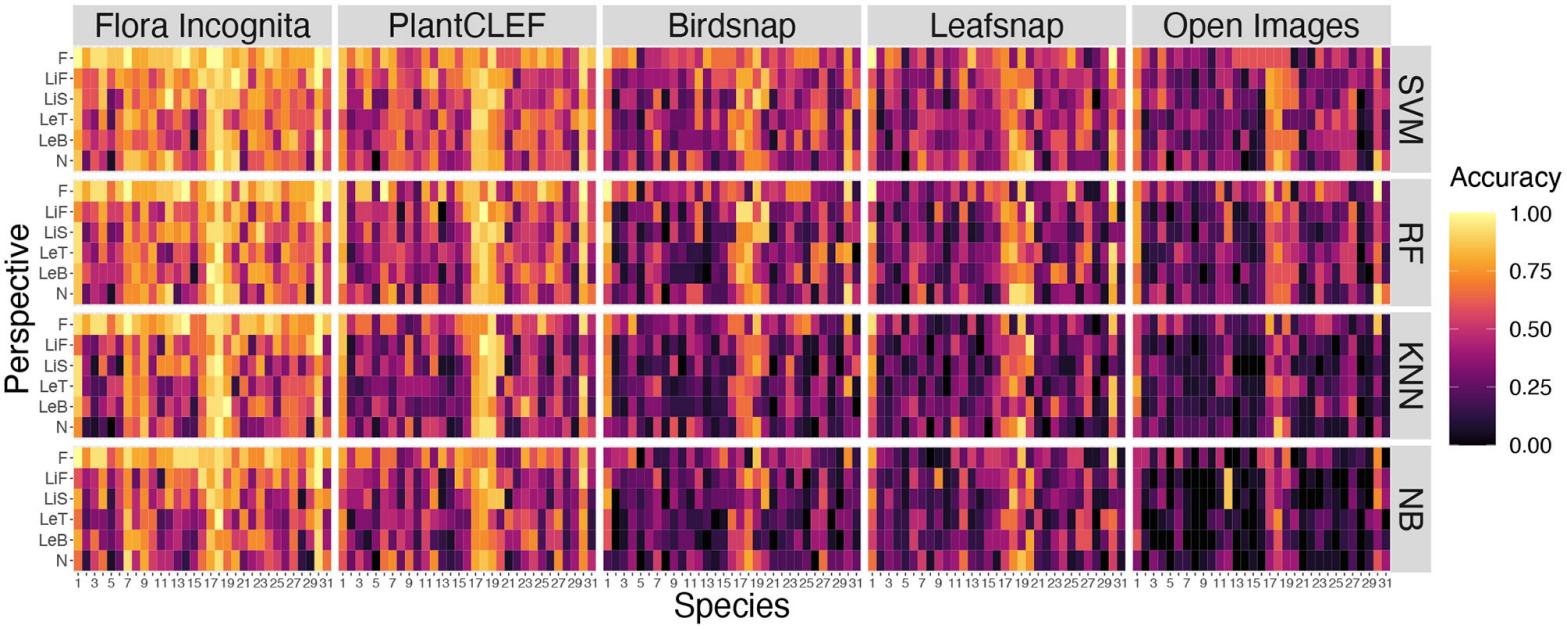
Comparison of accuracies per species for all single perspectives across all CNNs and all classifiers. Species codes are explained in Table 1.

**Figure 4:**
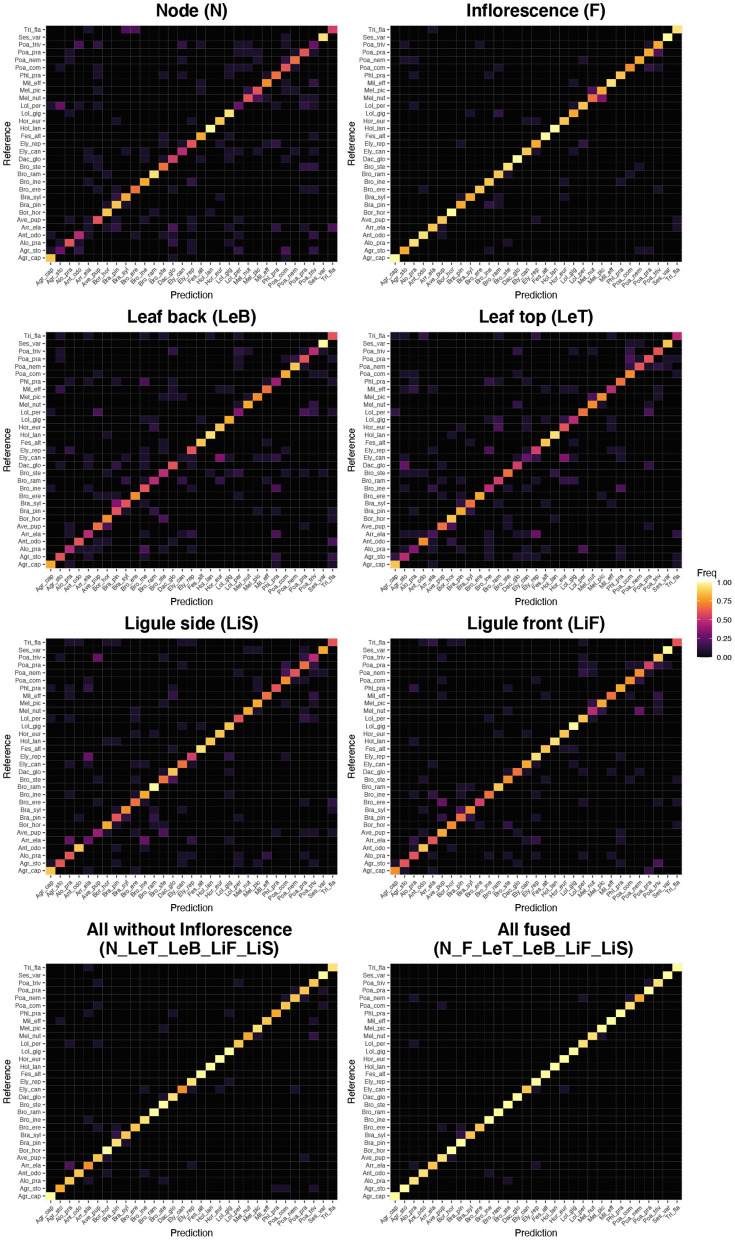
Confusion matrices (Reference vs. Prediction) for the best performing combinations of feature vectors and classifier (Flora Incognita neuronal network features combined with the SVM classifier) for the single perspectives, for the fusion of all perspectives but Inflorescence and for the fusion of all perspectives. Species abbreviations are explained in Table 1.

A few species are consistently recognized well based on a single image perspective regardless of feature extractor and classifier. Examples for this are *Agrostis capillaris, Festuca altissima, Holcus lanatus* and *Sesleria varia* (Figure 3). For some species, certain perspectives provide highly inaccurate information, e.g., the perspective node for *Arrhenatherum elatius*, which performs poorly across all feature extractors and classifiers (Figure 3). The fusion of all perspectives leads to very few misidentifications (Figure 4). The only species with three misidentifications out of 15 test observations is *Poa nemoralis* (two misidentifations: *Arrhenaterum elatius, Brachypodium sylvaticum, Bromus erectus*). 19 of 31 species are always correctly identified in all test observations. In general, the strip-like patterns that continues for many species throughout the entire array of feature descriptors and the classifiers in **Figure 6** indicate that the feature descriptors and the classifiers largely agree on which species are easy to classify and which are not. To compare the species accuracies across the different feature extractors we calculated relative accuracies as each species accuracy divided by the maximum of all species for this particular feature - classifier combination (**Figure 7**).

## 4. Discussion

The main goal of this study was to determine which image perspectives convey the most information to identify 31 species belonging to the Poaceae family. We found individual perspectives to be ranked in the following order: inflorescence (87.3%), ligule front (75.1%), ligule side (70.3%), node (64.9%), leaf back (63.9%) and leaf top (62.6%). Our results show that combining images taken from multiple perspectives further increases the success rate of identifying Poaceae species. Fusing all perspectives *via* sum rule we achieve an accuracy of 96.3%. Even without inflorescences, the 31 species under consideration in this study can still be identified with an overall accuracy of about 90% (Table 2). Combining only three perspectives (node, leaf top side, ligule frontal) turns out to be a reasonable compromise of taking as few pictures as possible while still achieving a high accuracy of 87.1% (Table 2).

Combining different perspectives has shown to be effective for improving overall accuracy before (Do et al., 2017; Rzanny et al., 2019; Nhan et al., 2020; Seeland and Mäder, 2021). A study using images of 12 Poaceae species from various perspectives found a maximum accuracy of 90% when all perspectives were combined (Rzanny et al., 2019). That study used a different approach and different perspectives compared to this study, e.g., an image of the ligule was not considered. The results of the present study, however, show that the frontal perspective of the ligule (LiF) is the second most informative one after the inflorescence (F). The ligule, and more generally the collar region of Poaceae, is also known to be of utmost importance for manual identification, since shape and size of the ligule, as well as presence, shape and hairiness of auricles are important distinctive characters (). Consequently, it is highly plausible that images of the ligule are also important for automated identification.

**Figure 5:**
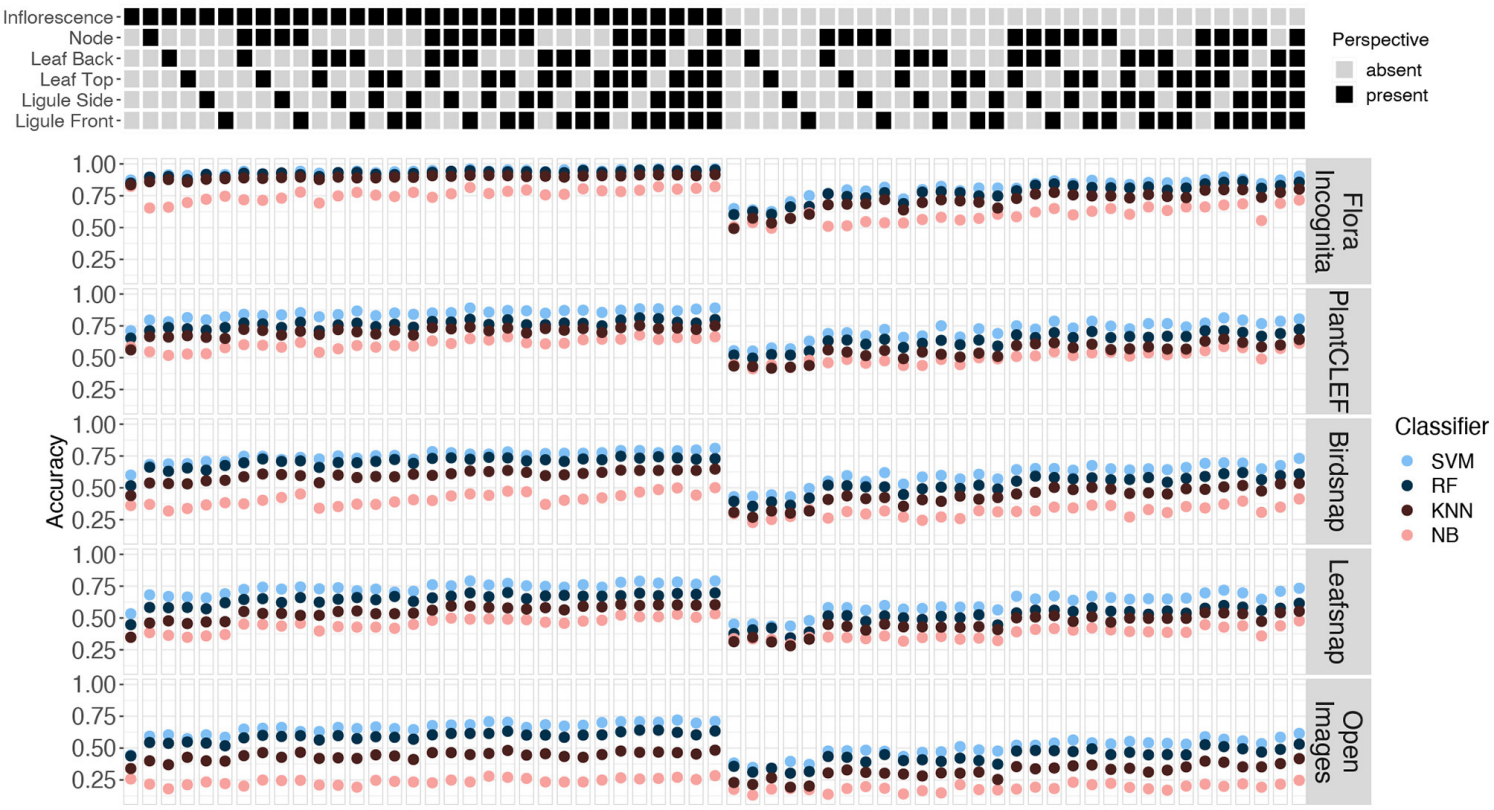
Accuracies achieved for combinations of image perspectives (columns) and feature extractors (rows). The presence or absence of the respective perspective in each combination combinations are indicated through the matrix at the top. The results are shown separately for each CNN with the classifiers color coded.

**Figure 6:**
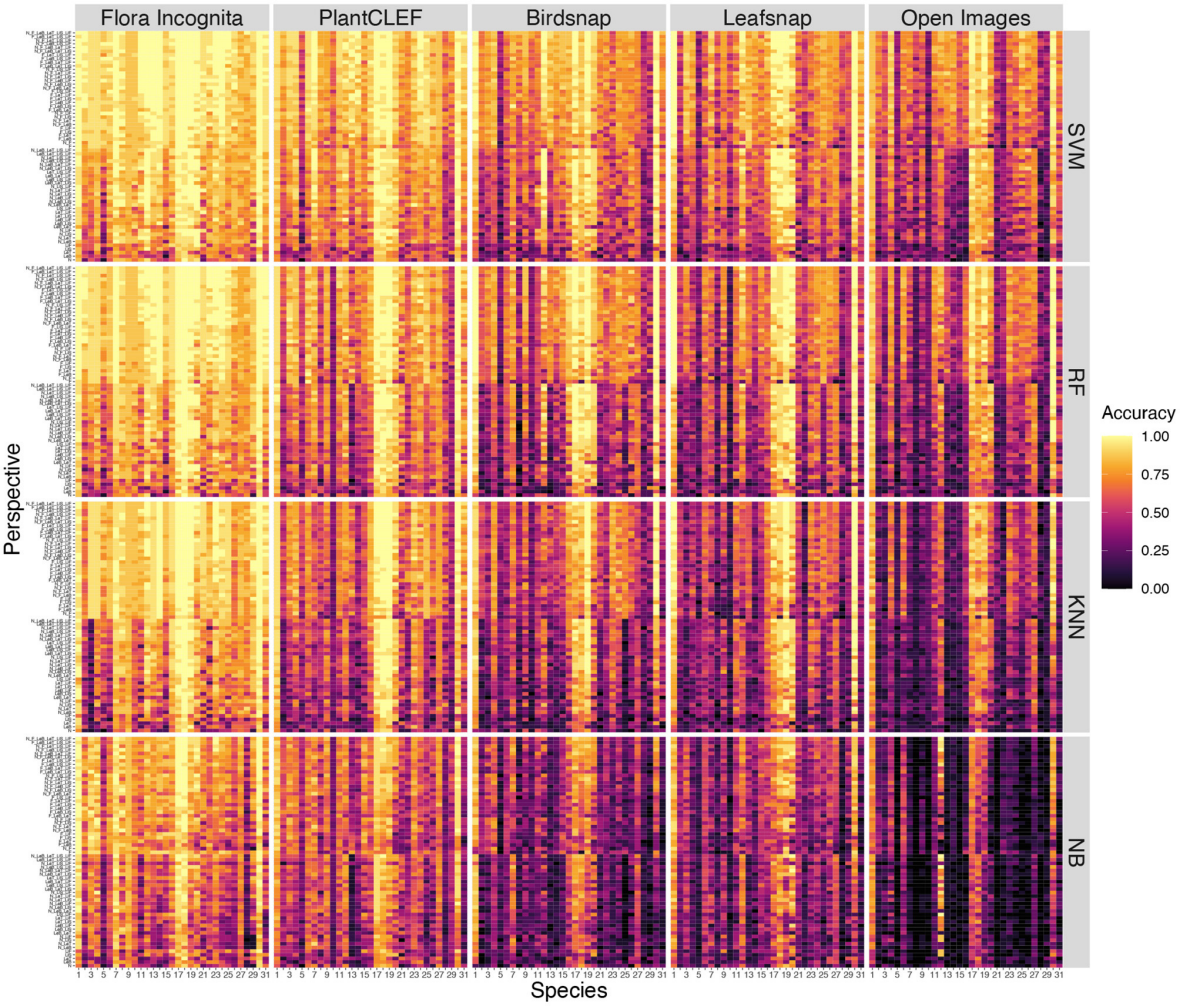
Accuracy per species for individual perspectives and all combinations for all feature extractor (colums) and classifiers (rows). The perspective combinations are subdivided into two sections: combinations containing inflorescences (upper section) and combinations that do not contain inflorescences (lower section). Within these sections the combinations are sorted along the number of perspectives combined. Species codes are explained in Table 1.

**Figure 7:**
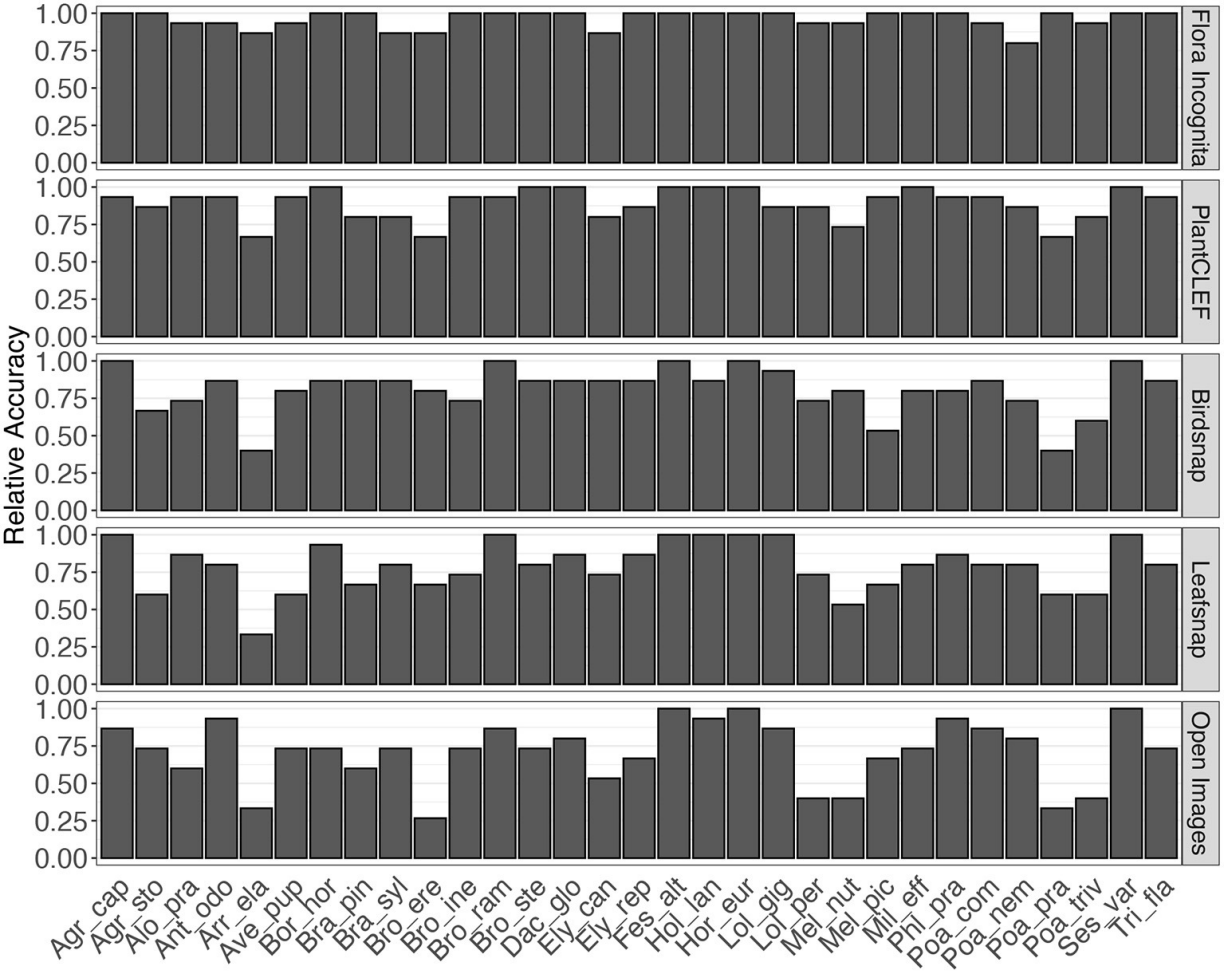
Relative accuracy per species for fusion of all perspectives and the best-performing classifier (SVM). Relative accuracy is calculated as species accuracy divided by the highest accuracy of all species for the particular feature/classifier combination.

**Figure 8:**
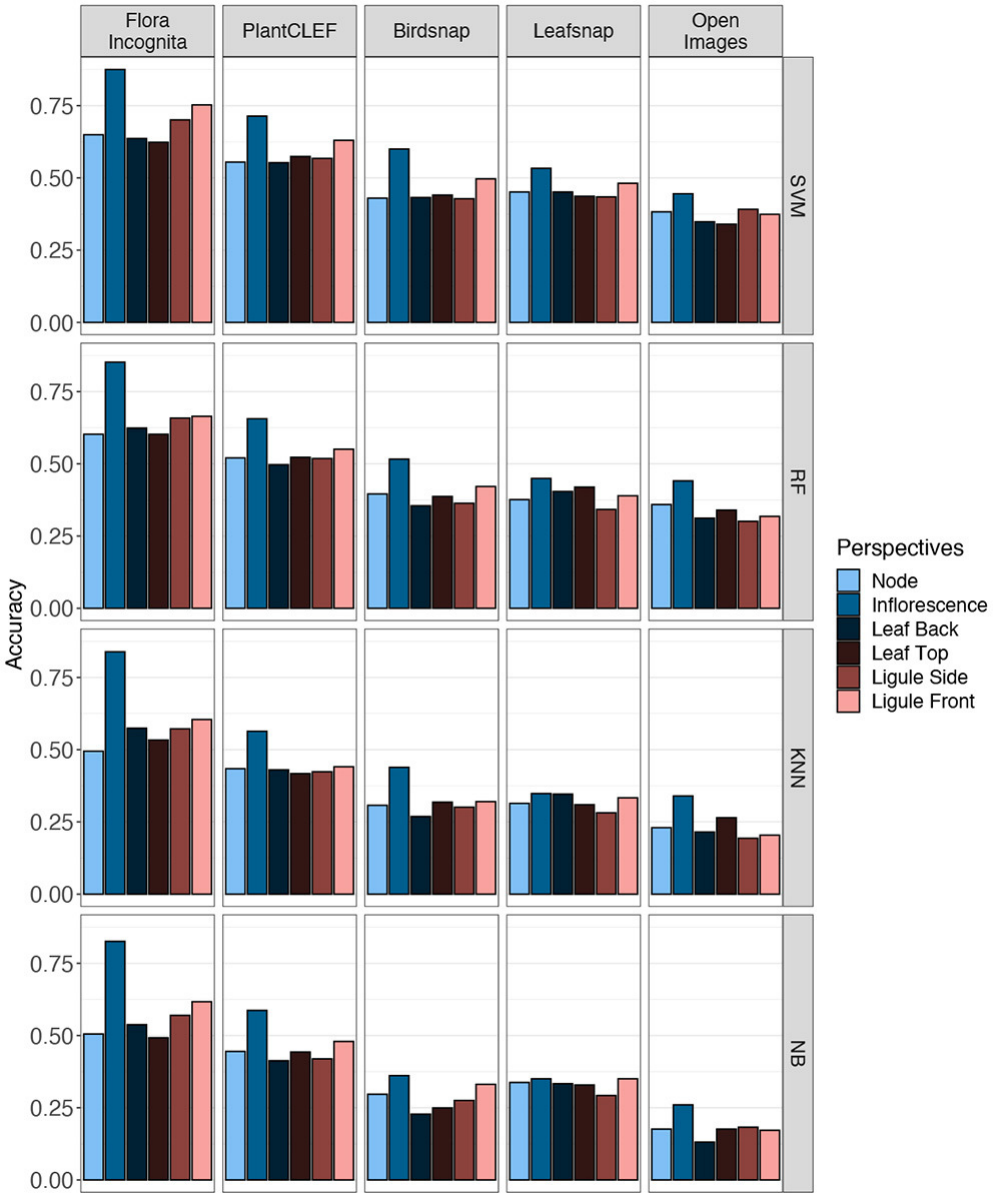
Accuracies achieved for the six single image perspectives. Values are shown for five different CNNs (columns) and four different classification algorithms (rows).

Different combinations of feature extractors and classifiers achieve a consistent ranking in the results for the same perspectives (Figure 5). This holds also true if accuracy is averaged across all species and also for individual species (Figures 6, 7), although there are larger differences in the absolute values. The example for the best performing classification algorithm (SVM) on all fused perspectives shows that none of the species that achieve less than 100% accuracy in the Flora Incognita feature extractor achieve 100% relative accuracy in any other feature extractor, indicating that all feature extractors more or less agree which species are difficult to identify and which are not (Figure 7). Similarly, there is a general agreement on the degree of importance of different perspectives for the identification of Poaceae species (Figures 3, 8). The fact that the ranking of classification accuracy among different classifiers is largely unaffected by the choice of the feature extractor is an indication that our results are not likely to be influenced by switching to another classification algorithm, therefore making our findings more generally valid by being largely classifier-independent. Even though we observe SVM to generally achieve highest accuracies, followed by RF, this may be explained by the fact that the features are derived from CNNs where they would originally be classified in a single fully-connected linear layer, making the problem more tractable for other linear classifiers such as SVMs. The differences in absolute values among feature extractors can be explained by the varying similarity of the domains and datasets used to train the original neuronal networks to our Poaceae images. While a number of grass species observations with partly detailed images are used to train e.g., the Flora Incognita network and the PlantCLEF network, such images did not or only marginally contribute to the training data of BirdSNAP and OpenImages. LeafSnap in turn is trained on cropped tree leaf images which do not share many features with the highly structured Poaceae images used in this study.

### 4.1. Limitations

Our study considers 31 Central European distributed species of Poaceae. However, there are more than 200 Poaceae species occurring in Germany (Müller et al., 2021) where all the images were taken. It is important to note, that the absolute accuracies for certain species are dependent on the number of species that need to be discriminated from each other. Here, we can only consider a small subset of all Poaceae species. Therefore, the achieved absolute accuracies need to be interpreted within the context of the considered species set. On the other hand, as Poaceae are characterized by a consistent morphological structure we think that the relative contribution of information content per perspective is transferable to other Poaceae species.

Our main aim is to show how identification accuracy within a certain group of species can be increased through an adequate choice of suitable perspectives. And our results imply that certain combinations of perspectives are consistently more informative across many different types of CNNs and classification algorithms.

In practice, it is difficult and requires some effort to take informative and focused images of specific Poaceae organs using a smartphone in the field. Poaceae have lineal leaves and often fuzzy, indistinctive inflorescences (Figure 1). Accordingly, plant parts only encompass small portions of the entire image while comparably large parts are covered by background. Additionally, some species have bristle-like, involute or even convolute leaves (e.g., *Festuca* spp., *Corynephoros canescens*, or *Nardus stricta*) which can render certain perspectives less useful and further diminish the leaf-background ratio of leaf images. Some taxa, e.g., within the *Festuca ovina* and *Festuca rubra* aggregates, are usually distinguished based on branching type of the tillers, leaf cross-sections or cytological differences (Stace et al., 1992; Dengler, 1998) which limits attempts to automatically identify taxa based on images below a certain threshold of taxonomic resolution. In other words, there are limits to certain taxa within Poaceae where a reliable automated identification based on macroscopic images is highly unlikely.

## 5. Conclusions

While Poaceae are a widespread, highly diverse and ubiquitous plant family that is shaping entire landscapes, they are very difficult to identify because its species closely resemble each other. Our observations show that, within a limited species pool even for those species, an accurate automated identification is possible as long as it is based on suitable images. Even if the most distinctive perspective, i.e., inflorescence, with which an overall identification accuracy of 96% can be achieved, is not available, accuracy only slightly decreases to 90%, which still leads to accurate predictions in most cases. These results imply that automated recognition of Poaceae is already useful for monitoring purposes or smart weeding approaches where the species pool is known. It remains to be explored further how reliable image recognition of Poaceae is in situations, where hundreds of species needs to be discriminated from each other. Poaceae represent only a single example of a species group that is difficult to identify. Other families such as Cyperaceae, Juncaceae, Equisetaceae, Cactaceae or certain genera such as *Alchemilla, Orobanche* or *Rosa* require their own unique perspectives of specific distinctive regions for a reliable identification. Many of these often apomictic and taxonomically challenging plant taxa (Dressler et al., 2017) have unique ecological requirements and are of great interest for monitoring and biodiversity conservation. It is therefore desirable to develop specific customized recording schemes for certain plant families to guide users of automated identification devices in taking images of these distinctive features during the identification process.

## Data Availability Statement

The datasets presented in this study can be found in online repositories. The names of the repository/repositories and accession number(s) can be found below: https://doi.org/10.6084/m9.figshare.16896496.v1.

## Author Contributions

MR, PM, JW, and HW: study conception and design. MR, AD, JW, and DB: data collection and preparation. MR and HW: analysis and interpretation of results and writing manuscript. MR and JW: visualization. PM and JW: funding. All authors provided critical feedback and approved the final manuscript.

## Funding

This study was funded by the German Ministry of Education and Research (BMBF) grant no. 01IS20062, the Federal Agency for Nature Conservation (BfN) with funds from the German Federal Ministry for the Environment, Nature Conservation, Building and Nuclear Safety (BMUB) grant nos. 3519685A08 and 3519685B08, and the Thuringian Ministry for Environment, Energy and Nature Conservation grant no. 0901-44-8652.

## Conflict of Interest

The authors declare that the research was conducted in the absence of any commercial or financial relationships that could be construed as a potential conflict of interest.

## Publisher's Note

All claims expressed in this article are solely those of the authors and do not necessarily represent those of their affiliated organizations, or those of the publisher, the editors and the reviewers. Any product that may be evaluated in this article, or claim that may be made by its manufacturer, is not guaranteed or endorsed by the publisher.
